# The regulation of yes-associated protein/transcriptional coactivator with PDZ-binding motif and their roles in vascular endothelium

**DOI:** 10.3389/fcvm.2022.925254

**Published:** 2022-07-22

**Authors:** Wen Zhang, Qian-qian Li, Han-yi Gao, Yong-chun Wang, Min Cheng, Yan-Xia Wang

**Affiliations:** ^1^School of Rehabilitation Medicine, Weifang Medical University, Weifang, China; ^2^Department of Rehabilitation Medicine, Affiliated Hospital, Weifang Medical University, Weifang, China; ^3^The Second Affiliated Hospital of Shandong University of Traditional Chinese Medicine, Jinan, China; ^4^School of Basic Medicine, Weifang Medical University, Weifang, China

**Keywords:** YAP/TAZ, endothelial cells, oxidative stress, inflammation, angiogenesis

## Abstract

Normal endothelial function plays a pivotal role in maintaining cardiovascular homeostasis, while endothelial dysfunction causes the occurrence and development of cardiovascular diseases. Yes-associated protein (YAP) and its homolog transcriptional co-activator with PDZ-binding motif (TAZ) serve as crucial nuclear effectors in the Hippo signaling pathway, which are regulated by mechanical stress, extracellular matrix stiffness, drugs, and other factors. Increasing evidence supports that YAP/TAZ play an important role in the regulation of endothelial-related functions, including oxidative stress, inflammation, and angiogenesis. Herein, we systematically review the factors affecting YAP/TAZ, downstream target genes regulated by YAP/TAZ and the roles of YAP/TAZ in regulating endothelial functions, in order to provide novel potential targets and effective approaches to prevent and treat cardiovascular diseases.

## Introduction

The vascular endothelium is a cell layer lining the internal surface of the vascular lumen (1). Endothelial cells can sense factors acting on the vascular inner wall, such as fluid shear stress, stretch stress, and extracellular matrix (ECM) hardness, and then release nitric oxide, prostacyclin, reactive oxygen species (ROS), and other vasoactive substances to maintain the normal function of blood vessels (2, 3). Yes-associated protein (YAP) and transcriptional co-activator with PDZ-binding motif (TAZ), two closely related transcriptional regulators in the classical Hippo signaling pathway, play a crucial role in organ growth, tissue regeneration, and tumor development through the regulation of diverse transcriptional factors (4–6). Recently, researchers have found that YAP/TAZ also play an indispensable role in regulating endothelial biological functions, including inflammation, oxidative stress, and angiogenesis (7–12). In the present review, we aim to describe the YAP/TAZ structural characteristics, summarize the factors regulating YAP/TAZ, and elucidate the downstream target genes regulated by YAP/TAZ and the effects of YAP/TAZ on vascular endothelial functions.

## Structural characteristics of yes-associated protein/transcriptional co-activator with PDZ-binding motif

**FIGURE 1:**
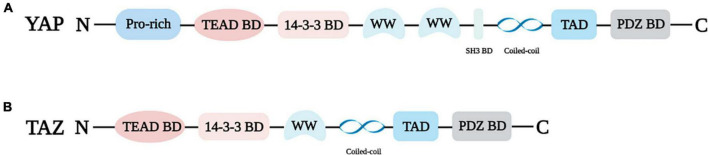
Structural properties of YAP **(A)** and TAZ **(B)**. Pro-rich: Proline-rich; TEAD BD: TEAD binding domain; 14-3-3 BD: 14-3-3 binding domain, WW: WW domain; SH3 BD: SH3 binding domain; PDZ BD: PDZ binding domain.

In 1994, Sudol identified and cloned the cDNA of a new protein that binds to the SRC homology 3 (SH3) domain of Yes proto-oncogene product through an anti-idiotypic antibody (13), and since then YAP has been discovered. Transcriptional regulators YAP and TAZ have quickly attracted the attention of researchers due to their important roles in cell growth and differentiation, tissue regeneration and repair, cancer, and cardiovascular diseases. YAP is mapped at chromosome 11q22 with a molecular weight of 65 kDa (14, 15). The N-terminal of YAP is connected to the proline-rich ligand. Because YAP lacks a DNA binding domain, it can only act as a transcriptional regulator *via* interacting with the TEAD binding domain or other transcription factors (16, 17). TEAD is a pivotal DNA binding platform of YAP and includes the 14-3-3 binding domain. The phosphorylation site of YAP at serine 127 (S127) was found to interact with 14-3-3 protein, resulting in the accumulation of YAP in the cytoplasm (18). YAP, also known as YAP1, contains eight splice isomers, and YAP1-1 and YAP1-2 are the two main isomers (15, 19, 20). The difference is that YAP1-1 has only one WW domain, while YAP1-2 contains two WW domains. The WW domain can identify the PPxY motif (proline/proline/any amino acid/tyrosine), which is present in a series of proteins known to be YAP/TAZ interactors (19). In addition, YAP harbors an SH3 binding domain that is located between the WW domain and coiled-coil domain (17). TAD is the transcriptional activation domain, and the PDZ binding domain is the C-terminal domain (Figure 1A).

TAZ, the paralog of YAP, was isolated as 14-3-3 binding protein, and it is located on chromosome 3q23-3q24 (14, 21). YAP and TAZ share several similar structures, but also possess distinctive structural features (Figure 1). The important shared structural features are the TEAD binding domain, WW domain, SH3 binding domain, Coiled-coil domain, and C-terminal transactivation domain. The main structurally distinctive feature is a proline-rich motif at the N-terminal end of YAP that is not conserved in TAZ (19) (Figure 1B).

YAP and TAZ are located both in the cytoplasm and nucleus. Phosphorylation of YAP/TAZ on multiple serine residues by LATS and other kinases, such as AKT and JNK contributes to YAP/TAZ inactivation and cytoplasmic accumulation (22, 23). Nevertheless, phosphorylation by c-Abl on YAP^*Y*357^ results in YAP/TAZ activation and the sequestration of YAP/TAZ into the nucleus (24, 25). The nuclear localization of YAP/TAZ plays a vital role in determining cell behaviors, including proliferation, differentiation, and migration.

## Regulatory factors of yes-associated protein/transcriptional co-activator with PDZ-binding motif

### Effect of mechanical stress on yes-associated protein/transcriptional co-activator with PDZ-binding motif

Studies have shown that YAP and TAZ are not only nuclear sensors in the Hippo pathway, but also signal carriers and amplifiers of mechanical stress in the extracellular microenvironment (26). YAP/TAZ can sense and distinguish diverse mechanical stress and trigger different biomechanical responses. Zhong et al. used a microfluidic perfusion device to demonstrate for the first time that YAP could respond to different magnitudes of shear stress (27). Subsequent studies found that the activity of YAP/TAZ could be regulated by different forms of shear stress, including laminar shear stress (LSS) and oscillatory shear stress (OSS) (28–30). On the one hand, by promoting the LATS1/2-dependent phosphorylation of YAP^*S*127^ in the Hippo pathway, LSS inhibited the activation of YAP to resist inflammation and maintain the stability in normal endothelial cells (28, 31). On the other hand, LSS could also down-regulate YAP activation through autophagy-dependent pathway to decrease the expression of pro-inflammatory genes and interrupt the formation of atherosclerosis plaque (32). For damaged vascular endothelium, LSS ameliorated endothelial functions by activating YAP/TAZ. For example, in the injury model of cardiac microvascular endothelial cells (CMECs), LSS increased the expression of platelet-endothelial cell adhesion molecule-1 (PECAM1) and phosphorylated endothelial nitric oxide synthase (p-eNOS). The increased PECAM1 and p-eNOS subsequently activated YAP to protect CMECs from ischemia reperfusion injury (33). However, when human umbilical vein endothelial cells (HUVECs) were exposed to OSS, it was observed that YAP/TAZ was activated and translocated from vascular actin to the nucleus. Meantime, the expression of pro-inflammatory factors, including intercellular adhesion molecule-1 (ICAM-1), vascular cell adhesion molecule-1 (VCAM-1), and interleukin-6 (IL-6) were increased (29, 34, 35), which led to vascular endothelial injury and atherosclerosis. A recent study also revealed that HUVECs in the microfluidic chip showed obvious nuclear translocation after being stimulated by OSS for 6 h. Moreover, the activation of YAP/TAZ in HUVECs induced a significant increase in the expression of molecules related to the occurrence and development of atherosclerosis, such as ICAM-1 and von Willebrand factor (36). In addition, when human adipose-derived microvascular endothelial cells were stimulated with 20% stretch stress based on a normal physiological condition, the nuclear to cytoplasmic ratio of YAP in the experimental group cells was significantly higher than that in the control group (37), which indicated that physiological stretch stress could also promote the activation of YAP. The above evidence shows that mechanical stress, including LSS, WSS and physiological stretch stress, could modulate the activity of YAP/TAZ. Specifically, LSS can promote YAP^*S*127^ phosphorylation and inactivate YAP, while OSS and physiological stretch stress are able to activate YAP. Exercise is an effective method to improve endothelial functions and maintain vascular homeostasis. Our previous study showed that pulsatile flow shear stress induced by moderate intensity exercise was a key factor in mediating vascular endothelial functions improvement (38). However, the underlying mechanism is not entirely understood. Therefore, whether exercise-induced blood flow shear stress can regulate arterial endothelial functions *via* modulating YAP/TAZ deserves further study.

### Effect of extracellular matrix hardness on yes-associated protein/transcriptional co-activator with PDZ-binding motif

Extracellular matrix (ECM) hardness is one of the important factors that determine cell adhesion and diffusion processes, which also has significant impacts on cell growth, migration, and differentiation. The activation of YAP/TAZ could be modulated by the hardness of ECM (39–41). In the early report, YAP activation was observed in breast epithelial cells under the hard matrix. Dupont et al. found that the hard substrate promoted the translocation of YAP/TAZ into the nucleus, whereas the soft substrate remained YAP/TAZ in the cytoplasm (26). Afterward, Shin and Mooney compared the content of YAP in human K-562 cells under different ECM hardness and observed that the expression level of YAP in cells under the hard matrix was higher than that under the soft matrix (42), which again confirmed the previous research results. Relevant studies also pointed out that the process of ECM hardness regulating YAP not only depended on Rho GTPase activity and actin cytoskeleton tension, but also relied on Src family kinases (39, 43, 44). Recently, Deng’s team found that the stiff substrate promoted the expression of focal adhesion kinase (FAK) and p-Paxillin, and then elevated the level of Rac1 in cells, contributing to the increase in cytoskeleton tissue stiffness. Subsequently, YAP was transferred to the nucleus, and the expression of target genes was up-regulated to promote the formation of endothelial tip cells (45). Similarly, Matsuo et al. confirmed that YAP activation was decreased in endothelial cells on soft substrate compared to cells on stiff substrate, and the YAP-Dll4-Notch signaling pathway was involved in modulating the effect of substrate stiffness on endothelial cell functions (46). Other studies have pointed out that the change in YAP activity induced by ECM hardness also plays a crucial role in cardiomyocyte regeneration. In addition, in the mouse myocardial infarction model, ECM protein Agrin activated YAP by up-regulating FAK and LRP4-MuSK and then promoted cardiomyocyte proliferation (47–49).

### Effect of drugs on yes-associated protein/transcriptional co-activator with PDZ-binding motif

A series of drugs, including anti-atherosclerotic and anti-neoplastic drugs, have previously been found to downregulate YAP/TAZ activity in endothelial cells (25, 29, 31, 50). Anti-atheroslerotic drugs, such as rosuvastatin, simvastatin, and lovastatin, can inhibit YAP/TAZ activation to ameliorate the occurrence and development of cardiovascular diseases (29, 31, 50, 51). For example, rosuvastatin markedly attenuated YAP expression on TNF-α treatment and then decreased ICAM1 and VCAM1 expression in HUVECs, playing anti-inflammatory and atheroprotective roles (50). Simvastatin treatment significantly suppressed YAP/TAZ activation to attenuate the disturbed flow-induced proliferation and inflammation (29, 31). Lovastatin decreased YAP/TAZ activation and diminished angiotensin II-induced cardiovascular fibrosis (52). Additionally, methotrexate, an anti-neoplastic drug, also markedly inhibited disturbed flow shear stress induced YAP/TAZ activation in an AMPK-dependent manner, and further reduced pro-inflammatory factor secretion and monocyte adhesion in HUVECs (35). Thus, inhibition of YAP/TAZ activation *via* drugs is a promising endothelial protection and athero-protective therapeutic strategy.

Bosutinib, a tyrosine kinase inhibitor, significantly decreased the level of the phosphorylation of YAP at tyrosine 357 (Y357) and YAP activation to alleviate endothelium injury and the development of atherosclerosis (25). Likewise, salvianolic acid B, harmine and tetramethylpyrazine, the extracts from the traditional medicinal plants, inhibited YAP nuclear translocation and activation, and thus played a potent atheroprotective role (8, 9, 53). These inhibitor and extracts from the traditional medicinal plants might serve as potential therapeutical candidates for improving endothelial function and cardiovascular diseases *via* regulating the YAP/TAZ pathway.

### Other factors regulating yes-associated protein/transcriptional co-activator with PDZ-binding motif

In addition to the above factors, YAP and TAZ are also regulated by glucose metabolism, hypoxia, and osmotic stress (41, 54–62). Under normal physiological conditions, YAP promoted glucose metabolism by up-regulating glucose transporter 3. Phosphorylation of YAP^*S*127^ increased when glucose metabolism was insufficient. On the contrary, in response to high glucose stimulation, YAP was activated and unregulated, and YAP activation led to vascular endothelial inflammation and increased monocyte adhesion (41, 54–58). In myocardial fibroblasts, high glucose promoted the increase of YAP expression in the nucleus by down-regulating p-MST1 and p-LATS1, resulting in inflammation, cell proliferation, and invasion (59). High expression of YAP/TAZ and VCAM-1 and vascular intima thickening were also observed in diabetic mice (54, 60). Besides, when the cells were under hypoxia, the production of 3-hydroxymethylglutaryl CoA reductase (HMGCR) increased. The up-regulated HMGCR suppressed the activation of LATS1/2 in the Hippo signaling pathway, and further promoted YAP nuclear accumulation and induced the increase in cysteine-rich angiogenic inducer 61 (CYR61) and connective tissue growth factor (CTGF) (61, 62). In addition, osmotic stress-induced the increase in phosphorylation of YAP^*S*128^ through NLK kinase localized YAP/TAZ in the nucleus (63).

Taken together, LSS, soft matrix, and abovementioned drugs and potential drugs promote YAP/TAZ inactivation and cytoplasm accumulation to resist inflammation and maintain vascular homeostasis (8, 25, 27, 28, 31, 46). Whereas, OSS, physiological stretch stress, hard matrix, glucose metabolism, hypoxia, and osmotic stress lead to YAP/TAZ activation and nuclear translocation, and further stimulate the expression of their downstream target genes to cause vascular endothelial injury and atherosclerosis (26, 29, 34, 35, 54–60).

## Regulation of yes-associated protein/transcriptional co-activator with PDZ-binding motif on downstream target genes

Accumulating evidence has shown that YAP/TAZ induce the expression of downstream target genes after binding with the transcription factors of the TEAD binding domain and then plays an vital role in angiogenesis, ECM remodeling and atherosclerosis by regulating cell proliferation and migration (7, 29, 39, 47, 64–68) (Table 1). In a study of ApoE^–/–^ mice fed with high-fat diets, an abnormal increase in YAP/TAZ expression was found in endothelial cells, as well as the augmentation of CYR61, CTGF and ankyrin repeat domain 1 (ANKRD1) contents. Moreover, YAP/TAZ activation induced the up-regulation of abovementioned genes and promoted the proliferation and migration of ECs, contributing to the thickening of common carotid artery wall and narrowing of vascular cavity in mice clearly observed by HE staining. In addition, in *in vitro* cell studies, overexpression of YAP/TAZ also increased the expression levels of the abovementioned genes. Therefore, these results confirmed that CYR61, CTGF, and ANKRD1 were upregulated by YAP/TAZ activation (25, 28, 29, 31, 35). It was also observed that the expression of angiopoietin-2 (Ang-2), a regulator of angiogenesis, decreased accordingly after the targeted knockdown of YAP, revealing that Ang-2 was also unregulated by YAP activation (7, 69, 70). Likewise, target genes such as hear shock protein A12B (HSPA12B), deleted-in-liver-cancer 1 (DLC1), microfibrillar-associated protein 5 (MFAP5), cell division cycle 42 (CDC42), and delta-like ligand 4 (DLL4) were modulated by YAP to further promote vascular germination or the formation of vascular reticular structure (64, 65, 71). In addition, YAP inactivation led to the decrease in expression levels of downstream genes, such as insulin-like growth factor binding protein 3 (IGFBP3) and diaphanous homology 3 (DIAPH3), and the down-regulated IGFBP3 and DIAPH3 destroyed ECM remodeling by inhibiting the increase in ECM hardness (39, 47). In addition, YAP activation reduced the expression of tumor necrosis factor superfamily member 10 (TNFSF10) to cause cells apoptosis (71, 72). In brief, YAP/TAZ activation up-regulate the abovementioned downstream target genes, including CYR61, CTGF, ANKRD1, to affect cells biological functions *via* regulating cells proliferation, migration and apoptosis.

**TABLE 1:** Related downstream target genes of YAP/TAZ.

Name (abbreviation)	Expression level	Functions	References
Cysteine-rich angiogenic inducer 61 (CYR61)	↑	CYR61 promotes ECs proliferation and migration, which leads to atherosclerosis	(28, 29, 31, 84)
Connective tissue growth factor (CTGF)	↑	CTGF arouses ECs proliferation and is involved in atherogenesis	(29, 31, 84)
Ankyrin Repeat Domain 1 (ANKRD1)	↑	ANKRD1 triggers the development of atherosclerosis by promoting ECs proliferation and migration	(29, 67)
Angiopoietin-2 (Ang-2)	↑	Ang-2 regulates the germination of new blood vessels and triggers angiogenesis	(7, 70)
Heat shock protein A12B (HSPA12B)	↑	HSPA12B plays an important role in promoting ECs proliferation and regulating endothelial angiogenesis after myocardial infarction	(86)
Deleted-in-Liver-Cancer 1 (DLC1)	↑	DLC1 is crucial for sprouting angiogenesis and vascular homeostasis	(65)
Microfibrillar-associated protein 5 (MFAP5)	↑	MFAP5 promotes the tube formation of ECs	(71)
Cell division cycle 42 (CDC42)	↑	CDC42 regulates endothelial tip cell migration and promotes vascular tubular structure and morphogenesis	(95)
Diaphanous homology 3 (DIAPH3)	↑	DIAPH3 is a positive regulator in inducing ECM remodeling in cancer-associated fibroblasts	(39)
Insulin-like growth factor binding protein 3 (IGFBP3)	↑	IGFBP3 accelerates the process of ECM remodeling *via* promoting an increase in extracellular matrix stiffness	(47)
Delta-like ligand 4 (DLL4)	↑	DLL4 ameliorates damaged vascular endothelium and boosts the production of germinating blood vessels	(64)
Tumor necrosis factor superfamily member 10 (TNFSF10)	↓	TNFSF10 is considered as a regulator involved in cell apoptosis	(71, 72)

Symbols: the expression levels of each target gene in this table was in the context of YAP/TAZ upregulation; ↑, increase; ↓, decrease.

## Effect of yes-associated protein/transcriptional co-activator with PDZ-binding motif on the biological functions of the vascular endothelium

### Yes-associated protein/transcriptional co-activator with PDZ-binding motif and inflammation

Atherosclerosis is the main inducer of cardiovascular diseases. Studies have confirmed that the activation of YAP/TAZ in endothelial cells plays an important role in the occurrence and development of atherosclerosis by promoting an inflammatory response (12, 67, 73, 74). Overexpression of YAP in an ApoE^–/–^ mouse model upregulated the inflammatory related factors in arterial endothelial cells, such as IL-6, VCAM-1 and IL-8, and thus increased the atherosclerotic plaque and lesion range in aortic arch (8, 33). Moreover, it was found that after activating tumor necrosis factor-α (TNF-α) in HUVECs, YAP/TAZ expression was increased and transferred into the nucleus. The up-regulation of YAP/TAZ further promoted the increase in VCAM-1 and ICAM-1 in HUVECs, leading to inflammation (75). In addition, YAP/TAZ also increased monocyte adhesion by stimulating the JNK signaling pathway, triggering an inflammatory response in HUVECs (31). Yang et al. down-regulated YAP/TAZ in endothelial cells through the application of salvianolic acid B and found that inflammatory related factors, such as IL-6, IL-1β, and TNF-α, were decreased significantly, which confirmed that inhibiting YAP/TAZ reduced the expression of inflammatory factors (9). YAP/TAZ also acted as important regulators of macrophage intervention in the pro-inflammatory response and participated in the development of atherosclerosis through macrophages (73). Many researchers believe that the decrease of YAP/TAZ in cells inhibits the expression of inflammatory factors. However, Lv et al. found that the expression of E-selectin and ICAM-1 in mouse lung endothelial cells and the number of adherent neutrophils in postcapillary venules increased in mice with endothelial-specific deletion of YAP (76). This study suggested that YAP knockout did not suppress the expression of inflammatory factors, but promoted the endothelial cells activation and inflammatory response in pulmonary endothelial cells, which was inconsistent with the aforementioned studies (76). The reason of difference results might due to the cells observed in Lv’s research was pulmonary endothelial cells, which was different from HUVECs or other types of endothelial cells used in other investigations.

### Yes-associated protein/transcriptional co-activator with PDZ-binding motif and oxidative stress

The dynamic balance between the oxidation and antioxidant system plays an important role in maintaining the homeostasis of the body. Under normal circumstances, the human body has a natural antioxidant system to fight against the oxidation system. When the production of superoxide anion, hydrogen peroxide, hydroxyl radical and other oxides in the body exceeds that of superoxide dismutase (SOD), glutathione, and other antioxidants, oxidative stress occurs (77). Oxidative stress is involved in the pathogenesis of atherosclerosis. Studies have shown that YAP participated in the regulation of oxidative stress, leading to the occurrence of atherosclerosis (78–80). In human aortic endothelial cells induced by ox-LDL, after down-regulating YAP, the expression of ROS was generally reduced. The decrease in ROS alleviated the endothelial injury caused by oxidative stress (11). Other findings showed that knockdown of YAP increased the expression of Rac1 in cells, and the up-regulation of Rac1 further caused excessive production of ROS, which eventually led to cell death related to autophagy (79, 81). Consistent with these, in *in vivo* animal experiments, under the conditions of YAP inhibitors or RNAi silencing, SOD expression content in rats was significantly decreased compared to the control group (58). Additionally, a study found that the activity of YAP was affected by ROS in breast cancer cells. When ROS production was reduced, intracellular YAP and JNK activation was attenuated accordingly, leading to mitochondrial dysfunction and apoptosis (10). The results of abovementioned investigations might suggest that YAP and ROS could promote each other’s activation or expression (10, 11, 76–78).

### Yes-associated protein/transcriptional co-activator with PDZ-binding motif and angiogenesis

Angiogenesis is a process of forming new capillaries from pre-existing blood vessels, which involves a series of events, including endothelial cells germination, branching, lumen formation, and remodeling into a functional perfusion vascular network (82, 83). The significant role of YAP in angiogenesis has been repeatedly reported. It was found that the expression of YAP was increased in the process of differentiation from endothelial progenitor cells to endothelial cells. YAP nuclear localization further activated the vascular endothelium and promoted neovascularization, indicating that YAP is closely related to angiogenesis (7, 40). Conversely, YAP deletion seriously hindered the formation of the vascular network structure in endothelial cells (84, 85). In the mouse model of myocardial infarction, YAP overexpression reduced myocardial injury by promoting angiogenesis, improving cardiac function, and elevating the survival rate (86, 87). However, in mice, endothelial-specific deletion of YAP/TAZ induced the reduction and deformity of filopodia at the vascular front and the decrease and disarranged distribution of tight and adherent junction proteins, leading to destruction of vascular barrier network (88). Thus, YAP was shown to be an essential factor in promoting angiogenesis and treating ischemic cardiovascular diseases. YAP binded to signal transducer and activator of transcription 3 (STAT3), resulting in the phosphorylation of STAT3 and the increase of STAT3 expression in the nucleus, which further activated downstream Ang-2 and accelerated angiogenesis (70, 85, 89). Similarly, the miR-205/YAP pathway depended on STAT3 to promote vascular germination and angiogenesis in HUVECs (90, 91). Other studies have found that the DLL4-Notch1 signaling pathway was closely related to angiogenesis. Inhibiting the DLL4-Notch1 signaling pathway promoted the expression of vascular endothelial growth factor receptor 2 (VEGFR2), and VEGFR2 regulated downstream Ang-2 by activating YAP to repair damaged vascular endothelium (46, 64, 92). The abovementioned evidence shows that YAP/TAZ play an important role in angiogenesis and are expected to become potential targets for the clinical treatment of pathological angiogenesis-related diseases.

## Conclusion and perspectives

**FIGURE 2:**
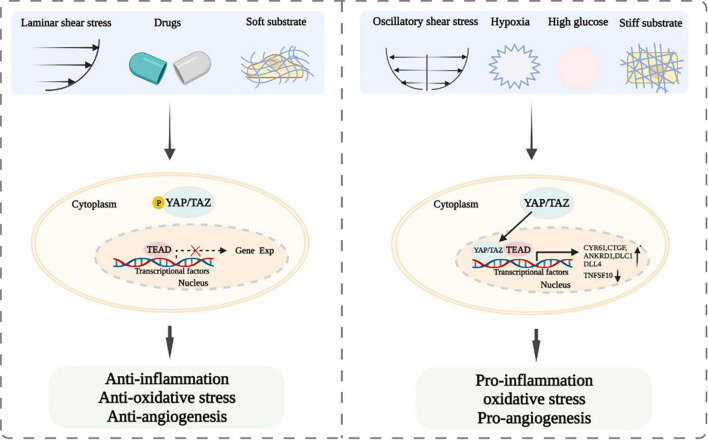
Schematic diagram of YAP/TAZ signaling and their modulation of endothelial functions after exposing to laminar and oscillatory shear stress, different drugs, soft and hard substrate, hypoxia or high glucose.

Endothelial dysfunction is one of triggers for the development of cardiovascular diseases (93, 94). YAP and TAZ are important downstream regulators of the Hippo pathway, which are involved in the regulation of vascular endothelial functions and play a prominent role in the development of cardiovascular diseases. Current studies have suggested that LSS, some drugs, and soft matrix induce YAP/TAZ inactivation and cytoplasm accumulation, resulting in the attenuating of inflammation and oxidative stress and the improvement of endothelial functions (25–29, 31). However, OSS, physiological stretch stress, and hard matrix cause YAP/TAZ activation and nuclear translocation, leading to vascular endothelial injury (34, 35, 41, 45) (Figure 2). Thus, modulating mechanical stress and matrix stiffness and using drugs might be served as treatment strategies for ameliorating endothelial functions. Our previous study showed that exercise-induced shear stress is a key factor to regulate endothelial function (38). However, the underlying mechanism is not entirely understood. Therefore, whether exercise-induced shear stress can improve arterial endothelial functions *via* modulating YAP/TAZ deserves further clarification. Furthermore, if YAP and TAZ mediate endothelial functions improvement under exercise-induced shear stress, whether the combined effects of exercise-induced shear stress and drugs would be achieve better synergistic effect in improving endothelial functions *via* YAP/TAZ regulation also need further study. In addition, glucose metabolism, hypoxia and osmotic stress can regulate YAP/TAZ and affect endothelial-related biological functions. However, how the abovementioned factors regulate YAP/TAZ and whether there are other downstream target genes modulated by YAP/TAZ are not well clarified. Accumulating evidence has indicated that YAP/TAZ play a central role in modulating biological functions of the vascular endothelium, including inflammation, oxidative stress, and angiogenesis (12, 67, 73–87). Whether there are other biological functions of the vascular endothelium affected by YAP/TAZ are also worthy to be further studied. Further studies of YAP/TAZ related biological functions of vascular endothelium and signal pathways will provide novel targets for the prevention and treatment of endothelial cells functions related cardiovascular diseases.

## Author contributions

Y-XW and MC designed the work. WZ, Y-XW, and Q-QL drafted the manuscript. H-YG, Y-CW, and MC revised the manuscript. All authors contributed to the article and approved the submitted version.

## Conflict of interest

The authors declare that the research was conducted in the absence of any commercial or financial relationships that could be construed as a potential conflict of interest.

## Publisher’s note

All claims expressed in this article are solely those of the authors and do not necessarily represent those of their affiliated organizations, or those of the publisher, the editors and the reviewers. Any product that may be evaluated in this article, or claim that may be made by its manufacturer, is not guaranteed or endorsed by the publisher.
